# Anthroponotic Cutaneous Leishmaniasis, Kabul, Afghanistan

**DOI:** 10.3201/eid0906.030026

**Published:** 2003-06

**Authors:** Richard Reithinger, Mohammad Mohsen, Khoksar Aadil, Majeed Sidiqi, Panna Erasmus, Paul G. Coleman

**Affiliations:** * London School of Hygiene & Tropical Medicine, London, U.K.; † HealthNet International, Peshawar, Pakistan

**Keywords:** Leishmaniasis, *Leishmania tropica*, humanitarian aid, anthroponotic, control, Afghanistan, dispatch

## Abstract

A prevalence survey in Kabul City showed that 2.7% and 21.9% of persons have active leishmaniasis lesions or scars, respectively. Incidence of disease was estimated to be 2.9% (29 cases/1,000 persons per year; 95% confidence interval 0.018 to 0.031). Disease was associated with age and gender; logistic regression analyses showed significant clustering of cases.

In Afghanistan, the majority of leishmaniasis cases are caused by *Leishmania tropica* (*1*), which is transmitted anthroponotically (i.e., humans are the reservoir) by the sandfly (*Phlebotomus sergenti)* (*2*). Anthroponotic cutaneous leishmaniasis (ACL) is generally characterized by large and/or multiple cutaneous lesions with a variable tendency to self-cure (*3*). Because of sandfly exposure, most lesions occur on the face, often leading to severe stigmatization in affected persons (e.g., women with lesions are often deemed unsuitable for marriage or raising children). Anecdotal reports suggest that because of the political instability and destruction of the housing and health infrastructure during the Mojahedin and Taliban regimes, *L. tropica* cases have increased during the past decade, with current World Health Organization (WHO) estimates of 200,000 ACL cases in Kabul alone (*4*). Also, because of several factors (the mass migration of *L. tropica–*infected [and infectious] Afghan refugees; the sporadic treatment of ACL cases by WHO and nongovernmental organizations; and the virtual absence of vector-control strategies), *L. tropica* has spread to areas where ACL was previously nonendemic (e.g., northwestern Pakistan) (*5*).

## The Study

Before developing an ACL-control strategy, we conducted a cluster-based, house-to-house survey in Kabul City between July and September 2001 to collect data on the extent of ACL. The leishmaniasis transmission season is between April and October. Each of the city’s 14 districts was divided into random clusters according to the district’s population size, for a total of 90 sample clusters; 30 neighboring households were surveyed in each cluster, with the first household selected at random. A team of medical staff diagnosed disease in household members on the basis of presence or absence of ACL lesions or scars, number of lesions, and date of lesion onset; members were interviewed to collect demographic data such as gender and age. Because of logistic constraints, parasitologic diagnosis of ACL lesions (e.g., microscopic examination or parasite culture) was not carried out. However, in Afghanistan, ACL-like skin lesions from other causes are rare, and our experience suggests that clinical diagnosis has a sensitivity and specificity of >80% and >90%, respectively (Reithinger et al., unpub. data). Written approval to conduct the study was obtained from the Afghan Ministry of Public Health. Informed consent was obtained from study participants; all persons with active cases surveyed were offered free antileishmanial treatment at the HealthNet International clinic.

Of 26,892 persons surveyed, 726 (2.7%) and 5,900 (21.9%) had active leishmaniasis lesions or scars, respectively. Of those persons with ACL lesions, the mean lesion number was 2.4 (range 1–50) and the mean lesion duration (to survey date) was 9.1 months (range 0.1–96). A total of 26,887 observations, with full disease and demographic records, from 2,683 households from the 90 sample clusters, were used in logistic regression analyses with a binary outcome variable (ACL lesion or scar).

Four variables were created to assess the distribution of leishmaniasis cases: the prevalence of active lesions in other members of the same household, the prevalence of scars in other members of the same household, the prevalence of active lesions in the nearest neighbor households, and the prevalence of scars in the nearest neighbor households. The nearest neighbor households were defined in terms of the survey protocol. In a given sample cluster, the nearest neighbor to household 1 was household 2; the nearest neighbors to household 2 were households 1 and 3; and so forth. The logistic regressions with robust standard errors (i.e., clustering of households to control for within-household correlations) were also adjusted for age (continuous to year of age at last birthday), gender, and sample cluster (categorical 90 levels). All analyses were conducted in STATA 7 (Stata Corporation, College Station, TX).

The regression analyses show that female patients are at significantly higher odds of having leishmaniasis lesions or scars (Table). Odds of disease were associated with age: elderly people are at slightly greater risk of having active lesions; and elderly persons are less likely to have leishmaniasis scars (the drop in scar prevalence in persons >12 years was significant, odds ratio: 0.994 (95% confidence interval [CI] 0.991 to 0.997), p<0.001). The analyses showed significant clustering of ACL within and between households (Table). A person’s probability of having an active lesion was increased greatly if other lesions appeared on persons in the same household (Figure, A) and also (but less so) if other scars occurred on persons in the same household. A person’s probability of having a scar was greatly increased if persons with active lesions or scars were present in the same household (Figure, B). Finally, a person’s probability of having a scar was greatly increased with the presence of persons with active lesions or scars in neighboring households (Figure, C). No significant clustering of ACL lesions occurred between households; however, the sample size was small for analyses. Overall, these findings are consistent with clustering of ACL transmission, including transmission in areas where previous transmission has occurred (because of association with scar prevalence). When maximum likelihood methods (*6*) are used, the average annual force of ACL infection (λ) was estimated to be 0.029 per year (29 cases/1,000 persons per year; 95% CI 0.028 to 0.031) over the past 12 years (Figure, D).

**Table:** Within- and between-household clustering of anthroponotic cutaneous leishmaniasis lesions and scars, Kabul City, Afghanistan^a^

Explanatory variables	Outcome variable^b^	
	Lesion	Scar	
**Within-household prevalence**	OR (CI)	OR (CI)	
Prevalence of lesions in other household members	132.3 (67.3 to 259.8) p<0.001	3.728 (2.799 to 4.964) p<0.001	
Prevalence of scars in other household members	1.988 (1.500 to 2.635) p<0.001	48.24 (41.79 to 55.68) p<0.001	
**Between-household prevalence**			
Prevalence of lesions in nearest neighbor households	2.323 (0.984 to 5.486) NS	1.585 (1.036 to 1.353) p<0.05	
Prevalence of scars in nearest neighbor households	1.376 (0.957 to 1.980) NS	1.184 (1.036 to 1.353) p<0.05	
**Other**			
Age	1.005 (1.000 to 1.009) p<0.05	1.013 (1.011 to 1.015) p<0.001	
Sex (female relative to male)	1.383 (1.177 to 1.626) p<0.001	1.186 (1.108 to 1.270) p<0.001	
Sampling area^c^	Chi square=213.6, d.f.=89 p<0.001	Chi square=330.8, d.f.=89 p<0.001	
**Overall model fit**	Pseudo R^2^ = 11.89% p<0.0001	Pseudo R^2^=22.72 p<0.0001	

^a^Abbreviations used: OR, odds ratio; CI, 95% confidence intervals; d.f., degrees of freedom; NS, not significant. ^b^The statistical model controlled for age (in years), sex, and sampling area (a total of 90 areas), while the standard errors were adjusted for sampling of persons at the household level. ^c^The overall significance of the variable is shown rather than the ORs for the 90 different sampling areas.

**Figure:**
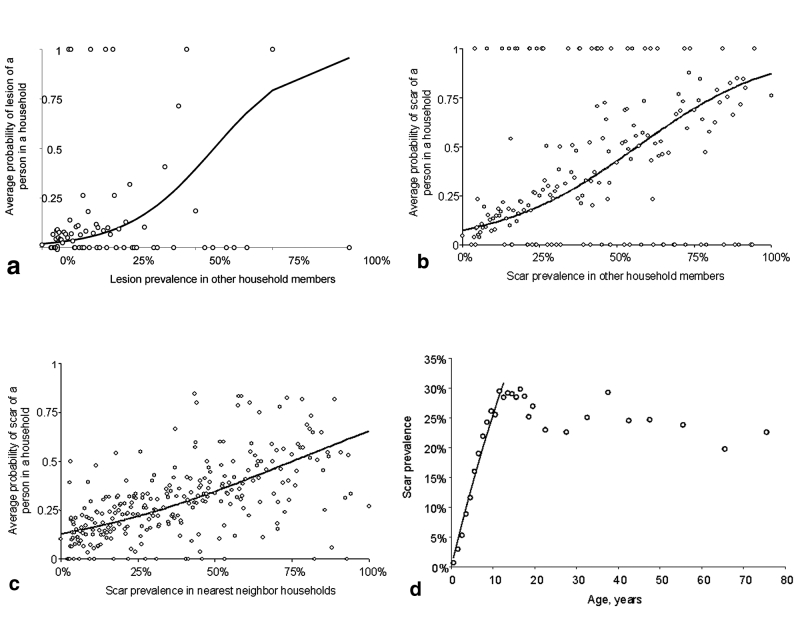
A, the average probability of having a lesion at different levels of lesion prevalence recorded among other members of the same household (open circles) and the unadjusted fit (solid line) from the logistic regression. B, the average probability of having a scar at different levels of scar prevalence recorded in other members of the same household (open circles) and the unadjusted fit (solid line) from the logistic regression. C, average probability of having a scar at different levels of scar prevalence in nearest neighbor households (open circles) and the unadjusted fit (solid line) from the logistic regression. D, force of infection, *λ,* can be estimated from the age-prevalence data, where the proportion, P, of persons with ACL at age *a* (where *a* is age at last birthday plus 0.5 years) is given by P(*a*) = 1-exp(-*λa*) (*6*). If one assumes that age-independent transmission started 12 years earlier (*1*), *λ* was estimated by maximum likelihood by using the observed age-prevalence data for children <12 y of age.

## Conclusions

Currently six clinics provide leishmaniasis diagnostic and treatment services in Kabul; an estimated 20% of the total 67,500 patients (based on the observed prevalence of 2.7% and a total 2.5 million population for Kabul) are diagnosed and treated. Whether this fact alone could explain the extent and duration of the leishmaniasis epidemic in Kabul is uncertain. Our analyses show that persons are at high risk for active ACL when a high proportion of persons with ACL scars are in the same or neighboring households. The likely explanation for this finding is that sandfly distribution and abundance are patchy but stable over time. The high prevalence of persons with active ACL in Kabul and the comparatively high ACL incidence show that ACL-control strategies (e.g., increasing the number of clinics providing treatment facilities or providing personal protection methods against sandflies) should be conducted soon. We demonstrate that a blanket-coverage ACL-control strategy is not necessary: transmission of this disease is focalized, and interventions (e.g., household insecticide spraying, insecticide-impregnated bednets or *chaddars*) (*5*) targeting households with a high proportion of persons with leishmaniasis lesions or scars or city districts containing a high number of high transmission clusters should have a major impact on transmission in Kabul.

The international donor community often considers ACL to be of peripheral importance (e.g., the disease was not included in the basic package of health services for Afghanistan) (*7*) because this disease has no impact on death rates and patient treatment costs (usually U.S.$15–200 [*8*]) are not recovered. Failure to implement a control strategy for this disease will likely lead to an increase in its impact and social stigmatization and represent further problems for a health infrastructure already crippled by 20 years of war.
